# Impact of Cardiac Arrhythmias on Acute Maternal Cardiovascular Outcomes in Pregnancy: A Systematic Review and Meta-Analysis

**DOI:** 10.3390/life16020278

**Published:** 2026-02-05

**Authors:** Antonios Siargkas, Alexandra Arvanitaki, Areti Faka, Efstratios Karagiannidis, Barbara Fyntanidou, Aikaterini Apostolopoulou, Themistoklis Dagklis, Apostolos Mamopoulos, Antonios P. Antoniadis, Nikolaos Fragakis, Ioannis Tsakiridis

**Affiliations:** 1 3rd Department of Obstetrics and Gynecology, Ippokrateion General Hospital, Faculty of Health Sciences, School of Medicine, Aristotle University of Thessaloniki, Konstantinoupoleos 49, 54642 Thessaloniki, Greece; asiargk@auth.gr (A.S.); dagklis@auth.gr (T.D.); amamop@auth.gr (A.M.); 2 2nd Cardiology Department, Ippokrateion General Hospital, School of Medicine, Aristotle University of Thessaloniki, Konstantinoupoleos 49, 54642 Thessaloniki, Greece; alexandra.arvanit@gmail.com (A.A.); aantoniadis@gmail.com (A.P.A.); fragakis.nikos@gmail.com (N.F.); 3 Department of Emergency Medicine, Ahepa University Hospital, School of Medicine, Aristotle University of Thessaloniki, St. Kyriakidi 1, 54636 Thessaloniki, Greece; stratoskarag@gmail.com (E.K.);

**Keywords:** pregnancy, cardiac arrhythmias, maternal mortality, major adverse cardiovascular events, systematic review, meta-analysis, cardio-obstetrics, atrial fibrillation, supraventricular tachycardia

## Abstract

Cardiac arrhythmias are prevalent complications in pregnancy, yet their precise association with acute maternal cardiovascular morbidity and mortality remains unclear due to heterogeneous evidence. This systematic review and meta-analysis evaluated the impact of maternal arrhythmias on acute cardiovascular outcomes. We searched Medline, Scopus, and Cochrane databases for observational studies comparing pregnant women with arrhythmias to those without. Random-effects meta-analyses were used to calculate pooled risk ratios (RR) for maternal mortality and major adverse cardiovascular events (MACE). Eleven studies comprising 76,028 pregnancies with arrhythmias and over 82 million controls were included. Analysis of data, primarily derived from large administrative cohorts with low absolute event rates, indicated that arrhythmias were significantly associated with increased all-cause maternal mortality (0.78% vs. 0.01%; RR 31.94; adjusted RR 8.91) and a composite of MACE (2.90% vs. 0.03%; RR 6.48). Supraventricular tachycardia and atrial fibrillation were associated with an increased likelihood of adverse outcomes. Notably, the relative risk of mortality and heart failure in women with arrhythmias versus controls was significantly higher in the general obstetric population around delivery than in women with known structural heart disease, suggesting a “sentinel event” phenomenon. Thromboembolic events were also 15 times more likely in the arrhythmia group. Cardiac arrhythmias during pregnancy are associated with substantial maternal morbidity and mortality. New-onset arrhythmias may warrant comprehensive cardiac evaluation, as they may unmask underlying pathology and precipitate severe hemodynamic compromise, particularly in women without prior cardiac history.

## 1. Introduction

Cardiac arrhythmias represent one of the most prevalent cardiovascular complications in pregnancy, posing substantial risks to both maternal and fetal health [1]. The incidence of arrhythmias in pregnancy has been rising over the past two decades [2]; this escalating trend is driven by a confluence of demographic and clinical changes, including advancing maternal age and the associated higher prevalence of cardiovascular comorbidities, a growing number of women with congenital heart disease surviving to childbearing age, and an increasing number of women with acquired heart disease [3]. Furthermore, the profound physiological, hemodynamic, and hormonal changes taking place in pregnancy may act as a catalyst and precipitate new-onset cardiac arrhythmias in apparently healthy women [4]. In addition, patients with a history of arrhythmias are at significant risk of arrhythmia recurrence during pregnancy [2].

The spectrum of arrhythmias encountered during pregnancy is broad; atrial arrhythmias are the most frequent ones, including atrial ectopic beats, supraventricular tachycardia (SVT), atrial fibrillation (AF) and atrial flutter (AFL), followed by premature ventricular contractions (PVCs), non-sustained ventricular tachycardia (NSVT), sustained ventricular tachycardia (VT) and ventricular fibrillation (VF) [5]. Until now, there has been heterogeneous evidence regarding the association of maternal arrhythmic events and maternal cardiovascular morbidity and mortality. Furthermore, there has been no published study pooling evidence on major adverse cardiovascular events related to arrhythmias; reported mortality risk ranges from a 7-fold increase for VT [6] and a 14-fold increase for atrial fibrillation [7] to as high as 100-fold for tachyarrhythmias, collectively [8]. Moreover, an increase in arrhythmia-related hospitalizations during pregnancy has been associated with an increased risk of both maternal and fetal complications, including maternal mortality [2]. Regarding the risk of heart failure among women with incident atrial fibrillation in pregnancy, one study found no significant association [9], while another reported an 8-fold higher risk [7]. Additionally, the physiological hypercoagulable state during pregnancy [10], may predispose women with atrial arrhythmias to an elevated risk of thromboembolic events, though the precise magnitude of this risk remains poorly defined [11]. Finally, there has been no pooled evidence on the impact of arrhythmias in women with structurally normal vs. abnormal hearts.

This systematic review and meta-analysis aimed to comprehensively summarize and synthesize all the available evidence on the association between maternal cardiac arrhythmias and the risk of acute adverse maternal cardiac outcomes in pregnancy and the puerperium. This study aims to provide a clearer, evidence-based understanding of the true impact of cardiac arrhythmias on maternal morbidity and mortality.

## 2. Materials and Methods

### 2.1. Protocol and Reporting

This systematic review and meta-analysis were conducted and reported in accordance with the Preferred Reporting Items for Systematic reviews and Meta-Analyses (PRISMA) guidelines [12]. The study protocol was established before the commencement of the literature search, outlining the objectives and methodology in detail, and was prospectively registered and published on the Open Science Framework (DOI 10.17605/OSF.IO/K6B4X). As this investigation relied exclusively on previously published data, approval from an ethical review board was not required.

### 2.2. Search Strategy and Study Selection

To address the research question regarding the association between maternal cardiac arrhythmias and adverse maternal cardiac outcomes, a comprehensive search strategy was developed using the PICO framework. A systematic search was performed across three major electronic databases: MEDLINE, Scopus, and The Cochrane Library. The search incorporated keywords and medical subject headings related to various types of cardiac arrhythmias (e.g., “atrial fibrillation,” “supraventricular tachycardia,” “ventricular tachycardia,” “ventricular fibrillation”) and terms related to pregnancy and is offered in our Supplementary Materials.

All identified records were imported into a reference management tool (Rayyan [13]) for duplicate removal. Subsequently, two reviewers (A.S. & A.F.) independently screened the titles and abstracts to identify potentially relevant studies. The full texts of the remaining articles were then meticulously evaluated to confirm their eligibility based on the predefined criteria. Any discrepancies between the reviewers were resolved through discussion or, if consensus could not be reached, by consulting a third reviewer (A.A.). Furthermore, the reference lists of all included articles were manually scanned to find any additional eligible studies.

### 2.3. Eligibility Criteria

Studies were considered eligible for inclusion if they were prospective or retrospective cohort studies, or case–control studies, investigating the association between maternal cardiac arrhythmias and adverse maternal cardiac outcomes. The study population consisted of pregnant women with or without a pre-existing structural heart disease. The exposure of interest was a diagnosis of any cardiac arrhythmia within one year before or during pregnancy and up to delivery including but not limited to AF/AFl, SVT, PVCs, NSVT/VT/VF, and pre-excitation syndromes. The control group comprised pregnant women without a diagnosis of the specific cardiac arrhythmia under investigation. Studies were excluded if they were case reports, conference abstracts, or did not provide sufficient raw data for analysis.

### 2.4. Outcomes

The primary outcomes were defined as all-cause maternal mortality and a composite of maternal major adverse cardiovascular events (MACE), including heart failure or pulmonary edema, cardiogenic shock, acute venous and/or arterial thromboembolic events (acute myocardial infarction, transient ischemic attack, ischemic or hemorrhagic cerebrovascular events and acute venous thromboembolism; deep vein thrombosis or acute pulmonary embolism) and cardiac arrest. Secondary outcomes consisted of the individual components of MACE.

The observation period for all primary and secondary outcomes was set as pregnancy and up to 6 months post-partum. A meta-analysis was performed for any outcome for which data were available from a minimum of three included studies.

### 2.5. Data Extraction

A standardized data extraction form was created in Microsoft Excel to ensure consistency. Two researchers (A.S. & A.F.) independently extracted relevant information from each eligible study. The extracted data included:

- Study Characteristics: First author, publication year, country of origin, and study design.
- Population and Clinical Details: Sample size, inclusion/exclusion criteria, specific type of cardiac arrhythmia, baseline patient characteristics (including the presence or absence of underlying structural heart disease and cardiovascular comorbidities), details on new onset versus recurrent arrhythmias, and information on management strategies such as the use of antiarrhythmic and anticoagulant medications or interventions like catheter ablation and other operations performed during pregnancy.
- Outcome Data: For dichotomous outcomes, raw data (number of events and total participants) were extracted to calculate risk ratios (RRs). Where available, adjusted effect measures were also collected, with a preference for data from adjusted statistical models.

Any disagreement during the data extraction phase was settled through discussion with a third reviewer (A.A) when necessary.

### 2.6. Quality and Risk of Bias Assessment

The methodological quality of the included studies was independently assessed by two reviewers (A.S. & A.F.) using the Newcastle–Ottawa Scale (NOS) [14]. Additionally, the risk of bias for each study was evaluated using the Quality In Prognosis Studies (QUIPS) tool [15]. Each of the six domains of the QUIPS tool was rated as having a low, moderate, or high risk of bias. Any disagreements were resolved through discussion with a third reviewer (A.A.).

### 2.7. Statistical Analysis

Statistical analysis was conducted to combine data for each outcome reported in three or more studies. The main effect measure was the risk ratio (RR) with its 95% Confidence Interval (CI), calculated from the raw data of the included studies. Due to the expected clinical and methodological diversity among the studies, a random-effects model using the DerSimonian and Laird method was applied to all meta-analyses.

Statistical heterogeneity was evaluated using Cochran’s Q test, with a *p*-value < 0.10 indicating significant heterogeneity. The magnitude of heterogeneity was quantified using the I^2^ statistic, with values greater than 50% considered to represent substantial heterogeneity. A *p*-value < 0.05 was deemed statistically significant for all pooled results. The potential for publication bias for the primary outcomes was assessed by visual inspection of funnel plots and by using Egger’s regression test, contingent on having at least 10 studies per meta-analysis.

### 2.8. Subgroup and Sensitivity Analyses

To provide a more thorough assessment, several pre-specified investigations were conducted:

Subgroup Analyses: Women were stratified in subgroups based on the type of arrhythmia: atrial Fibrillation/Flutter, SVT, VT, or a composite subgroup including “any type of arrhythmic event” for studies that did not provide a more granular classification. Furthermore, a secondary analysis was performed to assess the impact of underlying structural heart disease by comparing outcomes in women with known cardiac disease versus those with structurally normal hearts. In this analysis, studies that included all-comers without differentiating between these two cohorts were classified as “general obstetric population”.

Sensitivity and Adjustment Analyses: To assess the robustness of our findings, we conducted an analysis by pooling adjusted effect estimates. For any outcome, where at least three studies provided adjusted data, using multivariable regression or propensity score matching, an inverse variance random-effects model was used to pool this information and report an adjusted risk ratio (aRR). A second pre-specified sensitivity analysis based on excluding studies with a high risk of bias was not separately reported. In fact, quality assessment revealed that a high risk-of-bias rating was driven exclusively by the lack of adjustment for confounding factors. Therefore, the primary sensitivity analysis on adjusted effect estimates rendered the second analysis redundant and uninformative.

## 3. Results

### 3.1. Selection of Included Studies

**Figure 1:**
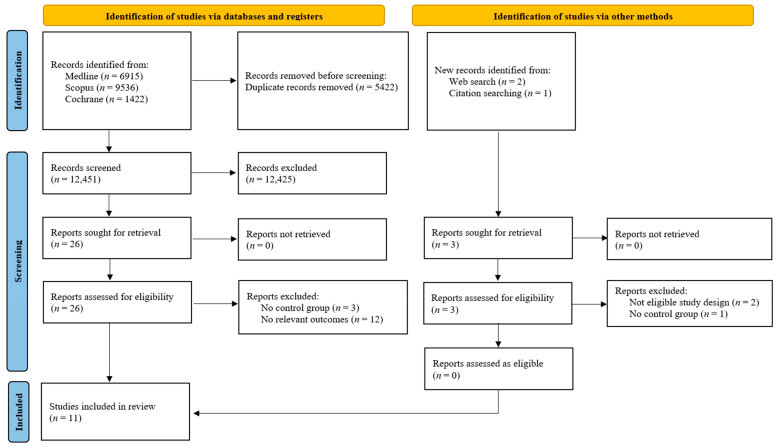
Study selection flowchart.

The initial literature search on 4 November 2025 identified a total of 17,873 records. Of these, 17,873 were sourced from electronic databases, Medline (*n* = 6915), Scopus (*n* = 9536), and Cochrane (*n* = 1422). After the removal of 5422 duplicate records, 12,451 unique articles underwent title and abstract screening. Three additional possibly relevant papers were identified through reference list searching for the initially screened articles. A total of 12,428 records were excluded from title and abstract screening. Upon full-text review, 18 reports were excluded for having an ineligible study design (*n* = 2), lacking a control group (*n* = 4), or not reporting relevant outcomes (*n* = 12). Finally, 11 studies [6,7,8,9,16,17,18,19,20,21,22] were deemed eligible and were included in the systematic review and meta-analysis. The detailed flow of the study selection process is illustrated in Figure 1.

The included studies reported on a total population of 76,028 pregnancies with any type of arrhythmia and 82,833,213 control pregnancies. The majority of the included studies were retrospective cohort studies (*n* = 8) [7,8,16,17,18,19,20,22], with the remaining being prospective cohort or case–control studies. Half of the studies (*n* = 6) [8,16,18,19,20,22] were conducted in the United States. There was heterogeneity in the population under study; some studies focused on arrhythmias in women with structurally normal hearts [16,17,21], while others specifically examined arrhythmic risk in patients with pre-existing cardiac conditions, i.e., rheumatic heart disease, congenital heart disease, or peripartum cardiomyopathy [6,7,9,19,22]. Furthermore, there were 3 studies that were conducted among women who were admitted for delivery and examined the impact of any arrhythmic event in the peripartum period [8,18,20]. A wide spectrum of arrhythmias was investigated, including atrial and ventricular arrhythmias. Most studies employed statistical methods such as multivariable regression or propensity score matching to adjust for confounders. Of the 11 studies included in the systematic review, 7 provided raw data [6,7,8,9,18,19,22] suitable for the primary meta-analyses. The study by Siochi et al. [20] was included as it provided a crucial adjusted effect measure for the sensitivity analysis. The remaining three studies, Bekiaridou et al. [16], Chou et al. [17] and Tong et al. [21], were eligible for the systematic review but were excluded from the quantitative synthesis (meta-analysis), as they reported zero events in both the study and the control group. This results in an indeterminate value, making it impossible to calculate the RR or its associated variance. Consequently, they could not be statistically pooled with the other studies. The detailed characteristics of the included studies are displayed in Table 1 and Supplementary Table S1.

### 3.2. Quality of the Included Studies

The methodological quality of the included studies was assessed using the NOS and is depicted in Table 2, with all 11 studies demonstrating high quality. Scores ranged from 7 to 9 out of a possible 9 points. Specifically, six studies achieved the maximum score of 9, three scored 8 points, and two scored 7 points. All studies performed well in the domains of Selection and Outcome, indicating robust case definitions, representative cohorts, and reliable outcome ascertainment. The primary source of potential bias was in the Comparability domain, where six studies did not receive both stars, suggesting they did not adequately control for important confounding factors in their design or analysis.

### 3.3. Quantitative Analysis

#### 3.3.1. All Cause Maternal Mortality

**Figure 2:**
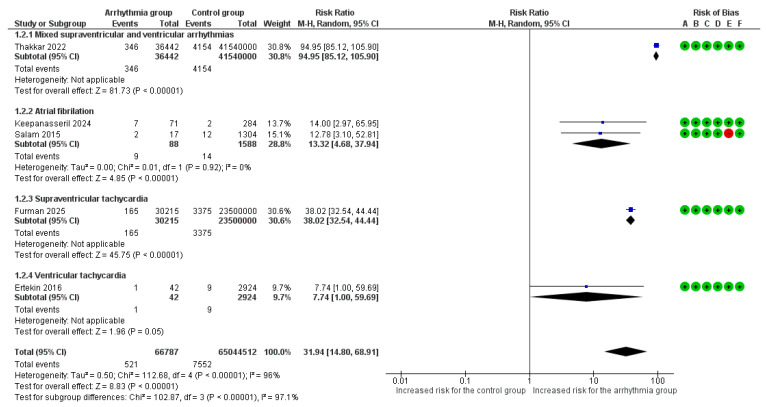
Forest plot illustrating the relationship between maternal cardiac arrhythmias and the risk of all-cause maternal mortality. Legend: CI, Confidence Interval; M–H, Mantel–Haenszel method [6,7,8,9,18].

Overall, 5 studies [6,7,8,9,18] reported maternal mortality as an adverse outcome. The absolute risk of maternal mortality was 0.78% (521/66,787) in the arrhythmia group compared to 0.01% (7552/65,044,512) in the control group. Overall, the presence of any cardiac arrhythmia was associated with a significantly increased risk of all-cause maternal mortality (RR = 31.94, 95%CI [14.80, 68.91], *p* < 0.001), with substantial heterogeneity observed across the studies (I^2^ = 96%) (Figure 2).

Μaternal mortality risk varied significantly across the different arrhythmia subgroups (*p* < 0.001). The highest risk was observed in the mixed arrhythmia cohort, which demonstrated a nearly 95-fold increase in mortality compared to women without any arrhythmic event (RR = 94.95, 95%CI [85.12, 105.90]). Pregnancies complicated by supraventricular tachycardia were associated with a 38-fold higher risk of death (RR = 38.02, 95%CI [32.54, 44.44]), while AF/AFL was associated with a 13-fold increased risk (RR = 13.32, 95%CI [4.68, 37.94]). Finally, ventricular tachyarrhythmia was linked to a borderline significant, nearly 8-fold increase in mortality (RR = 7.74, 95%CI [1.00, 59.69]).

A sensitivity analysis using adjusted estimates confirmed a significantly increased risk of maternal mortality (aRR = 8.91, 95%CI [3.22, 24.65]). Two subgroups had reported adjusted estimates; adjusted relative risk was higher in pregnancies complicated by AF/AFL (aRR = 17.30, 95%CI [5.84, 51.31]), followed by SVT (aRR = 4.68, 95%CI [2.94, 7.45]) (Supplementary Figure S1).

When stratified by underlying heart disease, the impact of arrhythmias on maternal mortality varied significantly (*p* = 0.02). No deaths were reported among women without a known cardiac condition either in the arrhythmia or the non-arrhythmia group. The relative risk of death associated with arrhythmias (versus no arrhythmias) was significantly higher in the general obstetric population in the peripartum period (RR = 60.19; 95% CI [24.05, 150.65]) than in the cohort of women with known cardiac disease (RR = 11.90; 95% CI [4.69, 30.20]). (Supplementary Figure S2).

#### 3.3.2. MACE

Overall, 7 studies [6,7,8,9,18,19,22] reported MACE as an adverse outcome. MACE occurred in 2.90% (1994/68,739) of pregnancies with arrhythmias versus 0.03% (21,749/65,052,662) of controls. The overall pooled analysis of all types of maternal arrhythmias demonstrated a statistically significant increase in the risk of MACE (RR = 6.48, 95%CI [1.05, 39.86], *p* = 0.04). Substantial heterogeneity was observed among studies (I^2^ = 100%).

**Figure 3:**
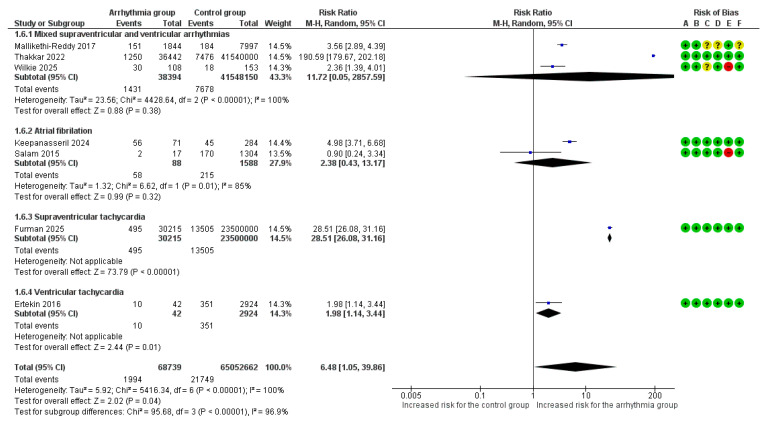
Forest Plot illustrating the association between maternal cardiac arrhythmias and major adverse cardiac events. Legend: CI, Confidence Interval; M–H, Mantel–Haenszel method [6,7,8,9,18,19,22].

Significant differences in the risk of MACE were identified among arrhythmia subgroups (*p* < 0.001) (Figure 3). SVT was associated with the highest risk, showing a nearly 29-fold increased risk of MACE (RR = 28.51, 95%CI [26.08, 31.16]). AF/AFL was associated with a 2.38-fold increased risk (RR = 2.38, 95%CI [0.43, 13.17]), while VT was associated with a nearly 2-fold increased risk (RR = 1.98, 95%CI [1.14, 3.44]). Mixed supraventricular and ventricular arrhythmias did not show a statistically significant association (RR = 11.72, 95% CI [0.05, 2857.59]).

In a sensitivity analysis for MACE using adjusted estimates, the overall risk was significantly elevated (aRR = 6.13, 95%CI [1.13, 33.40]). The highest adjusted risk among the available subgroups was observed for AF/AFL (aRR = 14.71, 95%CI [7.31, 29.61]), followed by mixed supraventricular and ventricular arrhythmias (aRR = 2.61, 95%CI [1.44, 4.73]) (Supplementary Figure S3).

In a subgroup analysis for MACE based on the underlying heart disease, the risk was significantly higher in the general obstetric population of pregnant women in the peripartum period with an arrhythmic event (RR = 73.73, 95% CI [9.68, 561.32]) compared to women with a known cardiac disease and arrhythmia (RR = 2.93, 95%CI [2.00, 4.30]) (*p* = 0.002) (Supplementary Figure S4). The subgroup of women with no heart disease had zero events in both the study and control groups, and inclusion in the analysis was not possible.

#### 3.3.3. Heart Failure or Pulmonary Edema

Overall, 5 studies [6,7,8,9,22] reported heart failure and/or pulmonary edema as an adverse outcome. The overall pooled analysis of all types of arrhythmias vs. no arrhythmic event did not demonstrate a statistically significant relative risk for heart failure and/or pulmonary edema (RR = 5.01, 95%CI [0.13, 196.17]). Extreme heterogeneity was observed among studies (I^2^ = 100%).

**Figure 4:**
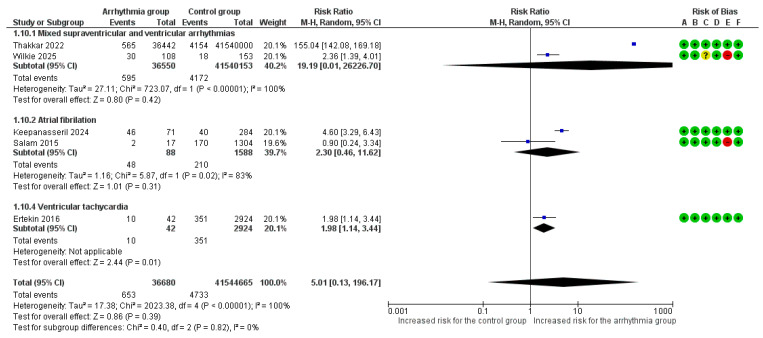
Forest Plot illustrating the association between maternal cardiac arrhythmias and heart failure. Legend: CI, Confidence Interval; M–H, Mantel–Haenszel method [6,7,8,9,22].

Manifestation of at least one VT event was associated with a significantly increased risk for heart failure and/or pulmonary edema (RR = 1.98, 95% CI [1.14, 3.44]). No differences were observed among various arrhythmia subgroups in the risk for heart failure and/or pulmonary edema (*p* = 0.82) (Figure 4).

**Figure 5:**
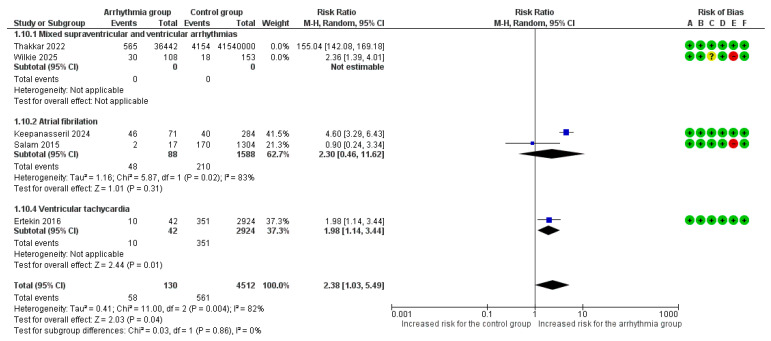
Forest Plot illustrating the association between maternal cardiac arrhythmias and heart failure after excluding mixed arrhythmias group due to extreme heterogeneity. Legend: CI, Confidence Interval; M–H, Mantel–Haenszel method [6,7,8,9,22].

Although both studies in the ‘mixed arrhythmias’ subgroup showed a positive association, the extreme disparity in effect sizes introduced significant heterogeneity (I^2^ = 100%) and unreliable confidence intervals. Consequently, we performed a sensitivity analysis excluding this subgroup (Figure 5). This refined analysis demonstrated a statistically significant risk of heart failure (RR = 2.38, 95% CI [1.03, 5.49], *p* = 0.04) with reduced heterogeneity (I^2^ = 82%) (Figure 5).

In a subgroup analysis for heart failure and/or pulmonary edema based on underlying cardiac disease, the risk was significantly higher when arrhythmias occurred in the general obstetric population in the peripartum period (RR = 155.04, 95%CI [142.08, 169.18]) compared to women with known cardiac disease (RR = 2.46, 95%CI [1.37, 4.43]), a statistically significant difference (*p* < 0.001) (Supplementary Figure S5).

#### 3.3.4. Cardiogenic Shock

**Figure 6:**
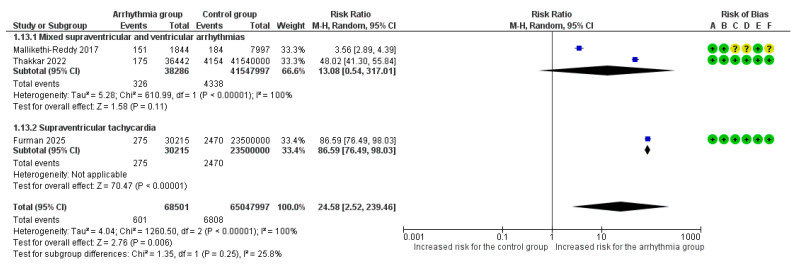
Forest Plot illustrating the association between maternal cardiac arrhythmias and cardiogenic shock. Legend: CI, Confidence Interval; M–H, Mantel–Haenszel method [8,18,19].

Overall, 3 studies reported cardiogenic shock as an adverse outcome [8,18,19]. Τhe overall pooled analysis demonstrated a significant 25-fold increased risk for cardiogenic shock in pregnant women with any arrhythmia vs. those without arrhythmic events (RR = 24.58, 95%CI [2.52, 239.46]), though with extreme heterogeneity across studies (I^2^ = 100%). The type of arrhythmia was not associated with a difference in risk for cardiogenic shock according to the subgroup analysis (*p* = 0.25) (Figure 6).

#### 3.3.5. Acute Thromboembolic Events

Overall, 5 studies [6,7,8,9,18] reported acute venous and/or arterial thromboembolic events as an adverse outcome. Τhe overall pooled analysis found a statistically significant association for pregnant women with arrhythmias (RR = 14.77, 95% CI [11.11, 19.62]), with low heterogeneity observed between studies (I^2^ = 28%).

**Figure 7:**
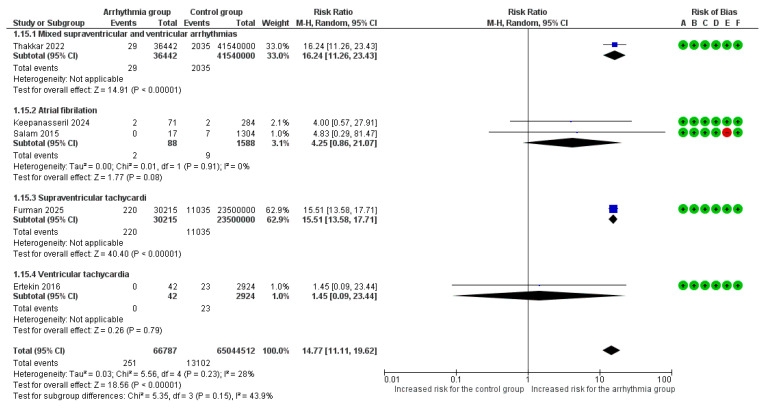
Forest Plot illustrating the association between maternal cardiac arrhythmias and thromboembolic events. Legend: CI, Confidence Interval; M–H, Mantel–Haenszel method [6,7,8,9,18].

In the subgroup analysis, this association was driven by mixed supraventricular and ventricular arrhythmias (RR = 16.24, 95%CI [11.26, 23.43]) and SVT (RR = 15.51, 95% CI [13.58, 17.71]). Νo significant variation in the risk of thromboembolic events was observed among different arrhythmia subgroups (*p* = 0.15) (Figure 7).

The results of the meta-analysis for the primary and secondary outcomes in the total cohort and in different arrhythmia subgroups are summarized in Table 3 and Table 4.

### 3.4. Publication Bias Analysis

**Figure 8:**
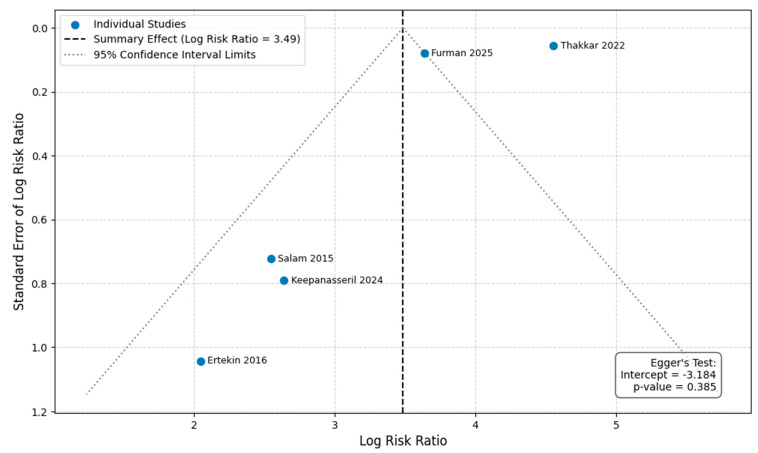
Funnel plot and Egger’s regression test result for overall mortality [6,7,8,9,18].

We performed Egger’s regression test to assess for funnel plot asymmetry and potential publication bias for our primary outcomes. The test was not statistically significant for either overall mortality (*p* = 0.385) or MACE (*p* = 0.061) (Figure 8 and Supplementary Figure S6). However, given the low statistical power of this test with fewer than 10 studies, these results cannot be used to reliably exclude the possibility of publication bias.

## 4. Discussion

### 4.1. Summary of Principal Findings

This meta-analysis provides a robust, quantitative synthesis of the risk for acute maternal cardiovascular outcomes in pregnancy or the puerperium associated with arrhythmic events occurring shortly before or during pregnancy. The presence of any cardiac arrhythmia in pregnancy was significantly associated with an increased risk of maternal mortality and a composite of MACE, including heart failure and/or pulmonary edema, cardiogenic shock, cardiac arrest, and acute venous and/or arterial thromboembolic events, even after accounting for potential confounding factors. AF/Afl was associated with a high risk for all-cause mortality and MACE, followed by SVT, after adjusting for confounders. Furthermore, the risk of all-cause mortality, MACE, and heart failure or pulmonary edema was substantially higher in the general obstetric population in the peripartum period compared to those with known cardiac disease.

### 4.2. Interpretation of Results

This study suggests an association between maternal cardiac arrhythmias and all-cause mortality in pregnancy and the puerperium, with a nearly 9-fold adjusted relative risk for death among women with arrhythmic events. This finding highlights the profound burden arrhythmias pose in pregnancy and their adverse impact on survival [6,7,8]. The exact cause of death has not been reported by all studies and the mechanisms vary substantially by arrhythmia subtype, reflecting their distinct pathophysiological impact within the proarrhythmic milieu of gestation [4].

Among the various arrhythmia types examined, women with SVT showed the highest unadjusted mortality risk. After adjusting for confounders, women with AF/AFL had a greater all-cause mortality risk, followed by those with SVT. The risk related to AF/AFL could be attributed to the following two major factors: hemodynamic instability caused by the loss of atrial systolic function and an increased likelihood of severe thromboembolism, which is further heightened by pregnancy-induced hypercoagulability [9,23,24,25]. SVT has been generally considered to have a safer clinical profile compared to other arrhythmias. However, this study highlights that new-onset SVT in pregnant women warrants serious attention and prompt management [26,27]. An SVT episode during pregnancy may be the first sign of underlying, previously unnoticed heart disease [4]. Although most SVT episodes are well-tolerated in non-pregnant individuals [28], the increased volume overload and heightened metabolic demands during pregnancy, can significantly lower the threshold for hemodynamic instability [26]. Rapid heart rates in SVT, which can surpass 200 bpm, dramatically shorten diastolic filling time, overwhelming the heart’s limited reserves and risking a critical drop in cardiac output and systemic blood pressure [26,29]. This study demonstrated the association of SVT with an 87-fold increased risk of cardiogenic shock, highlighting its potential to cause circulatory collapse in pregnant patients [26,29]. Finally, the high mortality risk linked to VT primarily results from its strong hemodynamic impact and its tendency to degenerate into ventricular fibrillation and cardiac arrest [30].

As expected, mortality risk was increased in women with arrhythmia and a known cardiac disease compared to those with arrhythmic events and structurally normal hearts, who had zero events. A pathological anatomical cardiac substrate increases arrhythmic risk during pregnancy. The exact mechanism of death in these women cannot be fully elucidated; sudden arrhythmic cardiac death, heart failure deterioration, or a thromboembolic event triggered by an arrhythmic event could be possible explanations [31]. A notable finding was that the risk of severe outcomes was significantly higher in the general obstetric population compared to women with known structural heart disease who developed arrhythmias. Several mechanisms may contribute to this disparity. First, women with known heart disease may benefit from “protective monitoring”; their arrhythmias, even minor ones, are anticipated and managed proactively. In contrast, women presumed to be generally healthy are at risk for critical diagnostic delays, as symptoms like palpitations or dyspnea may be dismissed as normal complaints of pregnancy, allowing severe pathology to progress undetected.

Beyond clinical management, this finding may also reflect inherent biases in administrative data. For an arrhythmia to be coded in the general population, it must typically be clinically significant enough to be noted and documented, potentially selecting for more severe, symptomatic, or sustained events, leading to selection bias. Conversely, in women with known heart disease, even minor, self-terminating arrhythmias are often documented as part of routine surveillance. Furthermore, the “general obstetric population” control group likely includes women with undiagnosed cardiac conditions, hypertension, obesity, or other risk factors not fully captured by coding, suggesting that the arrhythmia group may carry a higher burden of unmeasured comorbidities (residual confounding). Ultimately, these data highlight the exceptional vulnerability of the previously undiagnosed patient who presents with a new arrhythmia. This is particularly critical given that adverse cardiac events are more likely to manifest around delivery, a high-stress hemodynamic period, both in women with and without known cardiac disease [32].

Arrhythmias in pregnancy were also associated with a 6.5-fold increased risk for the composite outcome of MACE, which remained significantly elevated after adjusting for multiple confounding factors (aRR = 6.13). Adjusted sensitivity analysis provided additional clarity, identifying AF/AFL as carrying the highest risk for MACE among other arrhythmias, a finding consistent with its dual hemodynamic and thromboembolic burden in pregnancy [9,23,24,25]. Furthermore, the subgroup analysis based on underlying heart disease reinforced the “sentinel event” hypothesis: the MACE risk was almost three times higher in the general obstetric population compared to women with known cardiac disease. This disparity may imply that a new-onset arrhythmia in a woman presumed to be healthy is a potent red flag for acute decompensation, likely exacerbated by the diagnostic delays previously outlined [26]. However, more studies with clear reporting of the association of adverse cardiac events and arrhythmias in pregnant women during labor should be conducted to extract robust conclusions.

Regarding heart failure and pulmonary edema, the overall pooled analysis did not demonstrate a statistically significant association with arrhythmias. However, this finding may have been obscured by the extreme heterogeneity across the included studies. When analyzed by subgroup, a significant 2-fold increase in risk was identified specifically among women with episodes of ventricular tachycardia. Furthermore, a nearly 25-fold increased risk of cardiogenic shock in pregnancy was identified in women presenting with arrhythmic events before or during pregnancy, compared to women in sinus rhythm, an effect driven almost entirely by the SVT subgroup. This finding suggests that high-rate tachycardias may precipitate a state of circulatory collapse by critically impairing both ventricular filling and coronary perfusion [26,29].

Furthermore, this meta-analysis demonstrated a statistically significant 15-fold increased risk for acute venous and/or arterial thromboembolic events among pregnant women with arrhythmias vs. those without arrhythmic events. Due to lack of clinical data, the 2025 European Society of Cardiology (ESC) Guidelines for the management of cardiovascular disease and pregnancy propose that the decision of therapeutic anticoagulation in pregnant and postpartum women with new-onset or recurrent AF/AFl should be based on the same criteria (CHA_2_DS_2_-VA score) as in non-pregnant population [33]. However, given the profound biological plausibility of a “double-hit” mechanism, arising from pregnancy-induced hypercoagulability combined with atrial stasis, there has been a discussion among experts in favor of lowering the threshold for anticoagulation in pregnant women with AF [25]. Awaiting robust evidence, an individualized decision approach should be advised, taking into consideration personal history and other thromboembolic risk factors.

### 4.3. Clinical Significance of Findings

The results of this meta-analysis may have several clinical implications for the management of pregnant women. First and foremost, the findings suggest the importance of redefining what constitutes a “high-risk” pregnancy. The new onset of a sustained arrhythmia (SVT, AF/AFl or VT) in pregnancy should be considered a potential warning sign that warrants further evaluation by a pregnancy heart team with a comprehensive assessment for structural heart disease, in line with the multidisciplinary approach proposed by the 2025 ESC Guidelines for the management of cardiovascular disease and pregnancy [32] and the 2023 Heart Rhythm Society (HRS) expert consensus on the management of arrhythmias during pregnancy, even if not yet formalized as a distinct class-of-recommendation statement [5]. The ESC guidelines emphasize cardiovascular risk assessment in women with adverse pregnancy outcomes (Class I, Level B). Our findings support this statement, suggesting women with arrhythmias in pregnancy warrant extended surveillance. Therefore, any symptomatic or sustained arrhythmia (e.g., new onset sustained SVT or AF or VT) during pregnancy should trigger immediate referral to a cardio-obstetrics team and prompt, at a minimum, a 12-lead ECG and a transthoracic echocardiographic (TTE) examination, with a formal cardiology consultation, to rule out underlying pathology and risk stratify the patient, regardless of the perceived overall health status [32]. Cardiovascular assessment should be further expanded on identification of cardiovascular risk factors (e.g., arterial hypertension, obesity, diabetes mellitus, smoking, family history of cardiac inherited or acquired disease), investigation of undiagnosed coronary artery disease, cardiomyopathy or congenital heart disease. Apart from the fundamental TTE assessment, which is the first-line imaging modality, myocardial strain evaluation may provide additional information on cardiac function with prognostic significance [34,35]. Furthermore, cardiac magnetic resonance (CMR) may also be performed, without gadolinium, in case TTE is inconclusive to establish diagnosis and risk stratify the patient [36]. These women, irrespective of the underlying cardiac disease, warrant a continuous follow up throughout pregnancy and the post-partum, as there is an increased risk of arrhythmia recurrence and adverse pregnancy outcomes [37]. Furthermore, it is important to address symptoms in pregnancy such as palpitations, dizziness, and dyspnea with appropriate attention and care, rather than dismissing them as normal occurrences. These symptoms could indicate underlying health issues that require medical evaluation and should not be overlooked or minimized. Additionally, the results of this meta-analysis support the current ESC guideline and HRS statement emphasizing on early risk stratification, careful monitoring, and timely intervention in women with valvular and other structural lesions in whom arrhythmias occur during pregnancy.

### 4.4. Strengths and Limitations

This meta-analysis has several notable strengths. It is, to our knowledge, the most comprehensive quantitative analysis, focusing specifically on the maternal cardiac outcomes of arrhythmias in pregnancy. Furthermore, our sensitivity analysis using adjusted data from the primary studies strengthened the validity of our conclusions by demonstrating that the observed associations persist after accounting for key confounders. Our systematic search across multiple databases and adherence to PRISMA guidelines ensure the transparency and reproducibility of our methodology.

However, this study does have its limitations, which should be considered when interpreting the findings. Most of the included studies were retrospective in design, though the analysis also included prospective cohort and case–control studies. This variation in study design creates a mix of methodologies that may introduce different sources of bias and confounding. Second, there was significant statistical heterogeneity among studies, particularly for the composite outcomes of MACE and heart failure, that warrants careful interpretation. This high variance is primarily driven by the observational nature of the included studies and the inherent diversity of the populations analyzed. As detailed in Supplementary Table S1, there are substantial differences across studies regarding recurrent versus new-onset arrhythmias, the prevalence of comorbidities (e.g., hypertension, diabetes, thyroid disorders), and the extent of adjustment for confounding variables. Furthermore, variations in clinical management strategies, including the use of antiarrhythmic medications, anticoagulants, and catheter ablation procedures, likely contribute to the observed variance. Consequently, the pooled estimates likely represent an average of widely varying true effects across these distinct settings, making the precise magnitude of risk difficult to pinpoint with certainty. However, despite this variability, the direction of the effect remains consistent across studies, underscoring that while the exact risk estimate is context-dependent, the data support an adverse association between arrhythmias and maternal morbidity. Third, the number of studies available for certain arrhythmia subtypes, such as ventricular arrhythmias, and the outcomes reported were limited, affecting the precision of the pooled estimates. Furthermore, while we conducted a formal assessment of publication bias for the primary outcomes, the small number of studies limited the statistical power of these tests, and therefore, publication bias could not be reliably excluded. Additionally, despite the fact that we performed analyses using adjusted effect estimates to mitigate confounding, residual confounding from unmeasured or incompletely measured factors could not be ruled out, as the covariates used for adjustment varied significantly across studies. Finally, the granularity of data within the primary studies often precluded a detailed analysis of factors such as new onset versus recurrent arrhythmias, the timing of arrhythmia onset during gestation, antiarrhythmic medications used, or the need for catheter ablation, all of which could influence outcomes. Consequently, our ability to provide risk-stratified management guidelines based on arrhythmia duration or specific acute management strategies (pharmacological vs. electrical cardioversion) was limited.

## 5. Conclusions

This meta-analysis indicated that cardiac arrhythmias during pregnancy were associated with a substantial increase in maternal mortality and acute cardiovascular events. A critical finding was the identification of atrial tachyarrhythmias as a high-risk sentinel event, heralding severe hemodynamic compromise. The risk of severe maternal cardiovascular outcomes was higher in the general obstetric population compared to pregnant women with pre-existing cardiac conditions, underlying the importance of vigilant cardiac monitoring in pregnant women, particularly when new arrhythmias occur. This finding highlights the exceptional vulnerability of the previously undiagnosed patient who presents with a new arrhythmia at delivery, which is a period of increased hemodynamic stress, and requires prompt and thorough evaluation. These findings support, therefore, a lower threshold in clinical practice for comprehensive cardiac evaluation in symptomatic pregnant women, to ensure timely diagnosis and management of potential cardiac pathologies. To address current knowledge gaps, future research should prioritize prospective registries that systematically capture data on arrhythmia burden, frequency, duration, treatment pathways, and the specific temporal relationship between the arrhythmia onset and adverse maternal events.

## Figures and Tables

**Table 1:** Characteristics of the included studies.

Study	Period	Type	Country	Inclusion Criteria	Exclusion Criteria	Case Group	Control Group	Arrhythmias	Maternal Cardiac Outcomes	Follow Up Duration
Bekiaridou et al., 2024 [16]	2015–2022	Retro. Cohort	USA	• No known structural heart disease • Primiparous/multiparous without prior CS	History of: CMP, coronary artery disease, ischemic heart disease, myocarditis, VHD, CHD, RHD, or cardiac device	Pregnant women with documented SVT (before or during pregnancy) (*n* = 76) Age = 33.2 ± 4.8 y	Healthy pregnant women without SVT (4:1 ratio) (*n* = 304) Age = 32.1 ± 4.4 y	SVT	All-cause mortality	Pregnancy
Chou et al., 2023 [17]	2005–2020	Retro. Cohort	Taiwan	Singleton pregnancies at delivery/termination and ≥1 prenatal visit	• Age < 18 years • Incomplete records • Structural heart disease, sick sinus syndrome, atrioventricular block, HF, other tachyarrhythmias • Prior cardiac surgery/ablation/device	Pregnant women with PVC burden ≥ 1% on Holter up to 1 year before delivery or termination (*n* = 107) Age = 34.2 ± 4.3 y	Healthy pregnant women without PVCs (or other major diseases) (*n* = 214) Age = 34.0 ± 3.6 y	PVCs	All-cause mortality, post-partum heart failure, sustained VT	Up to 6 months after delivery or termination of pregnancy
Ertekin et al., 2016 [6]	2007–2013	Pro. Cohort	Multinational	Pregnant women with CHD, VHD, CMP, IHD, aortic pathology, or pulmonary hypertension	Arrhythmia in a structurally normal heart	Pregnant women with structural heart disease who developed ventricular tachyarrhythmia (symptomatic NSVT or VT) (*n* = 42) Age = 28.9 ± 5.7 y	Pregnant women with structural heart disease who did not develop ventricular tachyarrhythmia (*n* = 2924) Age = 29.3 ± 5.6 y	Ventricular Tachyarrhythmia (symptomatic NSVT or VT)	All-cause mortality, HF, thromboembolism, cardiac arrest, endocarditis	Up to 1 week post-partum
Furman et al., 2025 [18]	2016–2021	Retro. Cohort	USA	Pregnancy-related hospitalization with a diagnosis of SVT	N/A	Pregnant women hospitalized with SVT (*n* = 30,215) Age = 29.81 (29.65–29.97) y	Pregnant women hospitalized without SVT (*n* = 23,500,000) Age = 29.01 (28.96–29.05) y	SVT	All-cause in-hospital mortality, myocardial infarction, stroke, cardiogenic shock	During pregnancy-related hospitalization
Keepanasseril et al., 2024 [7]	2011–2015	Retro. Cohort	India	Pregnant women with RHD and AF diagnosed before or during pregnancy	N/A	Pregnant women with RHD and AF (*n* = 71) Age = 27.3 ± 4.5 y	Pregnant women with RHD without AF (*n* = 284) Age = 27.1 ± 4.7 y	AF	New-onset HF, thromboembolism, endocarditis, maternal mortality	Pregnancy and up to hospital discharge post delivery
Mallikethi-Reddy et al., 2017 [19]	2007–2012	Retro. Cohort	USA	Pregnant women ≥ 18 years hospitalized for PPCM	N/A	PPCM patients with arrhythmias (*n* = 1844) Age = 30.0 ± 7.24 y	PPCM patients without arrhythmias (*n* = 7997) Age = 30.06 ± 6.55 y	Various types of arrhythmias including VT, VF, bundle branch blocks, AF, AFl, AV block, SVT, WPW, premature atrial or ventricular complexes	In-hospital all-cause mortality, cardiogenic shock	During hospitalization for PPCM
Salam et al., 2015 [9]	2007–2011	Pro. & Retro. Cohort	Multinational	Pregnant women with structural heart disease (CHD, VHD, IHD, CMP)	Non-structural heart disease	Women with structural heart disease who developed AF/AFl in pregnancy (*n* = 17) Age= 32 ± 5.0 y	Women with structural heart disease who did not develop AF/AFl (*n* = 1304) Age = 30 ± 5.6 y	AF, AFl	All-cause mortality, HF, thromboembolism, endocarditis	Pregnancy and up to 6 months post-partum
Siochi et al., 2024 [20]	2016–2020	Retro. Cohort	USA	Patients > 18 years admitted for delivery	N/A	Patients with AF (chronic or new onset) (*n* = 7000) Age = 31.23 y	Patients without AF (general pop.) (*n* = 17,779,980) Age = 29.13 y	AF	In-hospital all-cause mortality	During hospitalization for delivery
Thakkar et al., 2022 [8]	2009–2019	Retro. Cohort	USA	Pregnant patients ≥ 18 years hospitalized for delivery	N/A	Hospitalized pregnant women with arrhythmia (*n*= 36,442) Age NR	Hospitalized pregnant women without arrhythmia (general pop.) (*n* = 41,540,000) Age NR	Various types of arrhythmias including SVT, AF, AFl, VT, VF	In-hospital all-cause mortality, stroke, cardiac arrest, acute HF, cardiogenic shock	During hospitalization for delivery
Tong et al., 2018 [21]	2010–2016	Pro. Case–Control	Canada	Pregnant patients referred for PVCs during pregnancy	• PVC burden < 1% • Spontaneous abortion < 20 wks • Structural heart disease • History of CVEs	(1) Pts with PVC > 1% (2) Pts with SVT (PVC < 1%) (PVC cases = 53, SVT cases = 53) Age NR	Low-risk normal pregnancy with no cardiac diagnosis (*n* = 53) Age NR	PVCs, SVT (different study subgroups)	Pulmonary edema, sustained VT, >10 runs of NSVT, stroke, cardiac arrest, cardiac death	Pregnancy and up to 6 months post-partum
Wilkie et al., 2025 [22]	2021–2025	Retro. Cohort	USA	Pregnant individuals with Ebstein’s anomaly	Other CHD	Pregnant patients with Ebstein’s anomaly who developed an arrhythmia (*n* = 108) Age = 32.0 ± 5 y	Pregnant patients with Ebstein’s anomaly without arrhythmia (*n* = 153) Age = 32.0 ± 6 y	Various types of arrhythmias, including AF/AFl, SVT, VA, PVCs, bradyarrhythmias	HF, thromboembolism, endocarditis, maternal mortality	Pregnancy

Abbreviations: AF, atrial fibrillation; AFl, atrial flutter; CHD, congenital heart disease; CMP, cardiomyopathy; CS, Caesarean section; CVEs, cardiovascular events; GDM, gestational diabetes mellitus; HF, heart failure; IHD, ischemic heart disease; NR, not reported; Obs., observational; PIH, pregnancy-induced hypertension; pop., population; PPCM, peripartum cardiomyopathy; Pro., prospective; PTB, preterm birth; PTL, preterm labor; PVC, premature ventricular complex; Retro., retrospective; RHD, rheumatic heart disease; SGA, small for gestational age; SVT, supraventricular tachycardia; VA, ventricular arrhythmia; VHD, valvular heart disease; VT, ventricular tachycardia; VF, ventricular fibrillation; wks, weeks; WPW, Wolff-Parkinson-White syndrome; y, years.

**Table 2:** Quality of the included studies based on the NOS.

First Author, Year	Study Type	S1	S2	S3	S4	C	O1	O2	O3	Total
Bekiaridou et al., 2024 [16]	Retrospective cohort	a *	a *	a *	a *	a *	a *	a *	a *	8
Chou et al., 2023 [17]	Retrospective cohort	b *	a *	a *	a *	a, b **	b *	a *	a *	9
Ertekin et al., 2016 [6]	Retrospective cohort	b *	a *	a *	a *	a *	b *	a *	a *	8
Furman et al., 2025 [18]	Retrospective cohort	a *	a *	a *	a *	a, b **	a *	a *	a *	9
Keepanasseril et al., 2024 [7]	Retrospective cohort	b *	a *	a *	a *	a, b **	a *	a *	a *	9
Mallikethi-Reddy et al., 2017 [19]	Retrospective cohort	b *	a *	a *	a *	a, b **	b *	a *	a *	9
Salam et al., 2015 [9]	Retrospective cohort	b *	a *	a *	a *	-	b *	a *	a *	7
Siochi et al., 2024 [20]	Retrospective cohort	a *	a *	a *	a *	a, b **	a *	a *	a *	9
Thakkar et al., 2022 [8]	Retrospective cohort	a *	a *	a *	a *	a, b **	b *	a *	a *	9
Tong et al., 2018 [21]	Prospective case–control study	a *	a *	b	a *	a *	a *	a *	a *	7
Wilkie et al., 2025 [22]	Retrospective cohort	b *	a *	a *	a *	a *	b *	a *	a *	8

Abbreviations: a, first answer according to Newcastle–Ottawa Scale (NOS); b, second answer according to NOS; S, selection; C, comparability; O, outcome; *, attribution of a star according to NOS; **, attribution of two stars according to NOS.

**Table 3:** Summary of pooled unadjusted risk ratios for primary arrhythmia subgroup analyses.

Outcomes	Overall Unadjusted RR [95% CI]	Mixed Arrhythmias Cohort RR [95% CI]	Atrial Fibrillation RR [95% CI]	Supraventricular Tachycardia RR [95% CI]	Ventricular Tachycardia RR [95% CI]
All-cause mortality	31.94 [14.80, 68.91]	94.95 [85.12, 105.90]	13.32 [4.68, 37.94]	38.02 [32.54, 44.44]	7.74 [1.00, 59.69]
MACE	6.48 [1.05, 39.86]	11.72 [0.05, 2857.59]	2.38 [0.43, 13.17]	28.51 [26.08, 31.16]	1.98 [1.14, 3.44]
Heart failure	5.01 [0.13, 196.17]	19.19 [0.01, 26226.70]	2.30 [0.46, 11.62]	-	1.98 [1.14, 3.44]
Cardiogenic shock	24.58 [2.52, 239.46]	13.08 [0.54, 317.01]	-	86.59 [76.49, 98.03]	-
Acute thromboembolic events	14.77 [11.11, 19.62]	16.24 [11.26, 23.43]	4.25 [0.86, 21.07]	15.51 [13.58, 17.71]	1.45 [0.09, 23.44]

**Table 4:** Summary of pooled adjusted risk ratios for primary arrhythmia subgroup analyses.

Outcomes	Overall Adjusted RR [95% CI]	Mixed Arrhythmias Cohort aRR [95% CI]	Atrial Fibrillation aRR [95% CI]	Supraventricular Tachycardia aRR [95% CI]	Ventricular Tachycardia aRR [95% CI]
All-cause mortality	8.91 [3.22, 24.65]	-	17.30 [5.84, 51.31]	4.68 [2.94, 7.45]	-
MACE	6.13 [1.13, 33.40]	2.61 [1.44, 4.73]	14.71 [7.31, 29.61]	-	-

## Data Availability

Not applicable.

## Supplementary Materials

The following supporting information can be downloaded at: https://www.mdpi.com/article/10.3390/life16020278/s1, Supplementary Figure S1. Forest Plot of the Association Between Maternal Cardiac Arrhythmias and All-Cause Maternal Mortality, Using Adjusted Estimates; Supplementary Figure S2. Forest Plot of the Subgroup Analysis by Underlying Cardiac Disease for the Outcome of All-Cause Maternal Mortality; Supplementary Figure S3. Forest Plot of the Association Between Maternal Cardiac Arrhythmias and Major Adverse Cardiac Events, Using Adjusted Estimates; Supplementary Figure S4. Forest Plot of the Subgroup Analysis by Underlying Cardiac Disease for the Outcome of Major Adverse Cardiac Events; Supplementary Figure S5. Forest Plot of the Subgroup Analysis by Underlying Cardiac Disease for the Outcome of Heart Failure; Supplementary Figure S6. Funnel plot and Egger’s regression test result for MACE; Supplementary Table S1. The rest of the characteristics for the included studies; Supplementary material S1. PRISMA 2020 checklist; Supplementary material S2. Search strategy.
