# Supplementary material for: Impact of Cardiac Arrhythmias on Acute Maternal Cardiovascular Outcomes in Pregnancy: A Systematic Review and Meta-Analysis
Source: Life (Basel). 2026 Feb 5;16(2):278. doi: 10.3390/life16020278 (PMC12941843; doi:10.3390/life16020278)
Supplement: Supplementary file 1 [file life-16-00278-s001.zip › S2. Search strategy broad.pdf]

All searches were performed on 04/11/25.

Pubmed

((pregnancy[MeSH Terms]) OR pregnant[tiab] OR gestation[tiab]) AND ((Arrhythmias, Cardiac[MeSH Terms]) OR "cardiac arrhythmia*"[tiab] OR (Atrial Fibrillation[MeSH Terms]) OR "atrial fibrillation"[tiab] OR (Atrial Flutter[MeSH Terms]) OR "atrial flutter"[tiab] OR (Tachycardia, Supraventricular[MeSH Terms]) OR "supraventricular tachycardia"[tiab] OR (Pre-Excitation Syndromes[MeSH Terms]) OR "pre-excitation syndrome"[tiab] OR (Atrial Premature Complexes[MeSH Terms]) OR "atrial ectop*"[tiab] OR "atrial premature"[tiab] OR (Ventricular Premature Complexes[MeSH Terms]) OR "ventricular ectop*"[tiab] OR "ventricular premature"[tiab] OR (Tachycardia, Ventricular[MeSH Terms]) OR "ventricular tachycardia"[tiab] OR (Torsades de Pointes[MeSH Terms]) OR "Torsade de Pointes"[tiab] OR "Torsades de Pointes"[tiab] OR (Bradycardia[MeSH Terms]) OR bradycardia[tiab] OR (Heart Block[MeSH Terms]) OR "atrioventricular block"[tiab])

6,915 results

Scopus

TITLE-ABS-KEY ((pregnancy OR pregnant OR gestation) AND ("cardiac arrhythmia*" OR "atrial fibrillation" OR "atrial flutter" OR "supraventricular tachycardia" OR "pre-excitation syndrome" OR "atrial ectop*" OR "atrial premature" OR "ventricular ectop*" OR "ventricular premature" OR "ventricular tachycardia" OR "Torsade de Pointes" OR "Torsades de Pointes" OR bradycardia OR "atrioventricular block"))

9,536 results

Cochrane

((pregnancy OR pregnant OR gestation) AND ("cardiac arrhythmia*" OR "atrial fibrillation" OR "atrial flutter" OR "supraventricular tachycardia" OR "pre-excitation syndrome" OR "atrial ectop*" OR "atrial premature" OR "ventricular ectop*" OR "ventricular premature" OR "ventricular tachycardia" OR "Torsade de Pointes" OR "Torsades de Pointes" OR bradycardia OR "atrioventricular block")

1,422 results
