# Supplementary material for: Impact of Cardiac Arrhythmias on Acute Maternal Cardiovascular Outcomes in Pregnancy: A Systematic Review and Meta-Analysis
Source: Life (Basel). 2026 Feb 5;16(2):278. doi: 10.3390/life16020278 (PMC12941843; doi:10.3390/life16020278)

Supplementary Figure S1. Forest Plot of the Association Between Maternal Cardiac Arrhythmias and All-Cause Maternal Mortality, Using Adjusted Estimates.

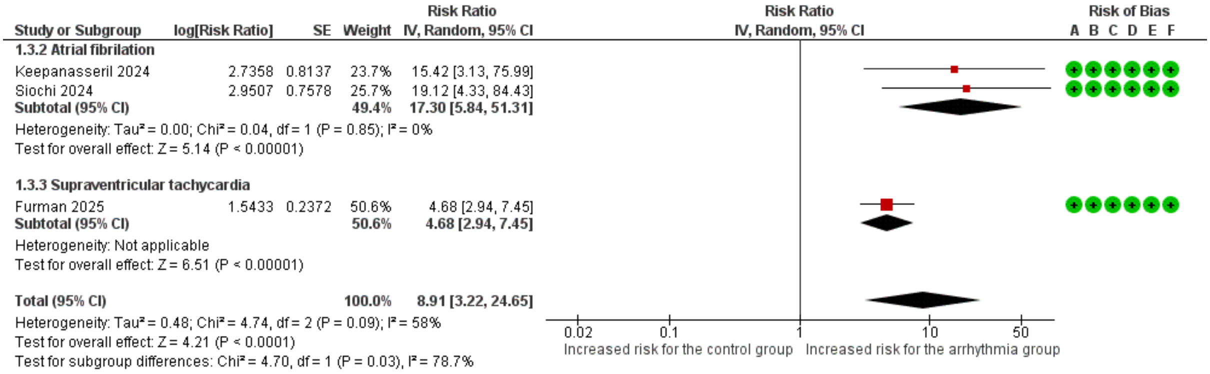

Supplementary Figure S2. Forest Plot of the Subgroup Analysis by Underlying Cardiac Disease for the Outcome of All-Cause Maternal Mortality.

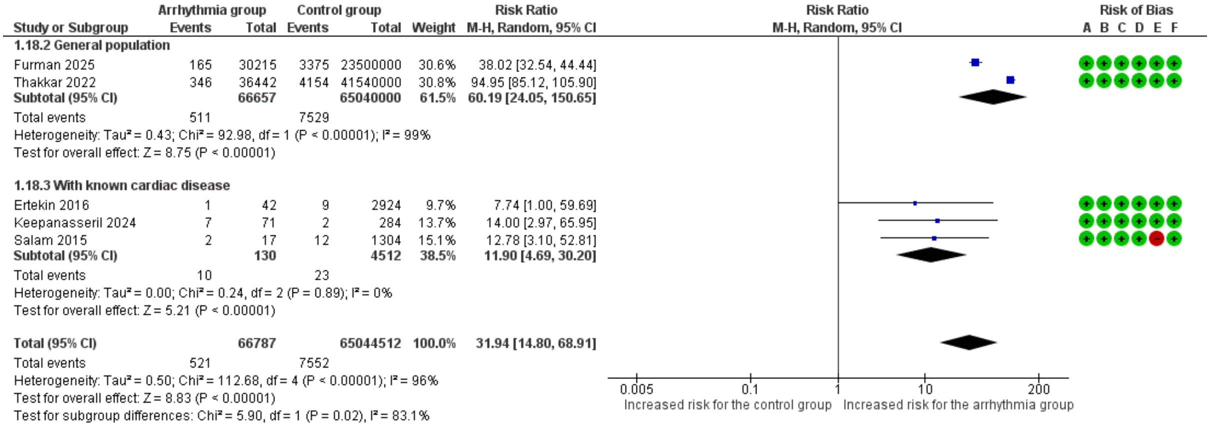

Supplementary Figure S3. Forest Plot of the Association Between Maternal Cardiac Arrhythmias and Major Adverse Cardiac Events, Using Adjusted Estimates.

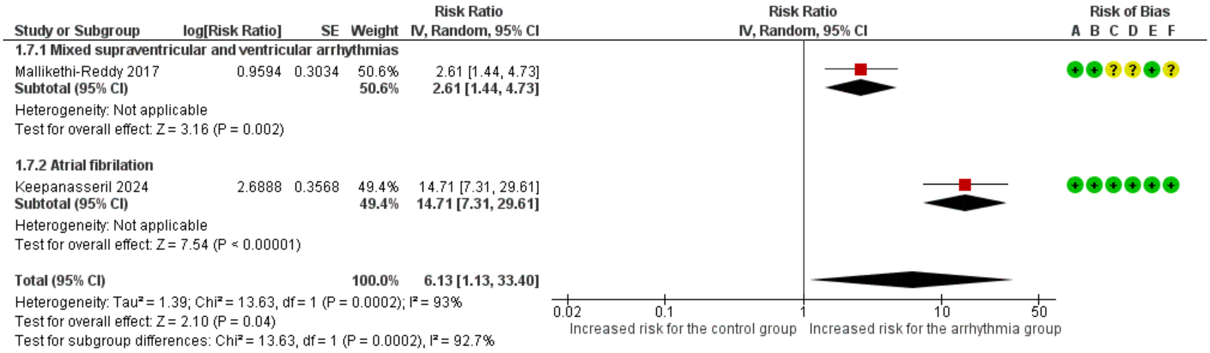

Supplementary Figure S4. Forest Plot of the Subgroup Analysis by Underlying Cardiac Disease for the Outcome of Major Adverse Cardiac Events.

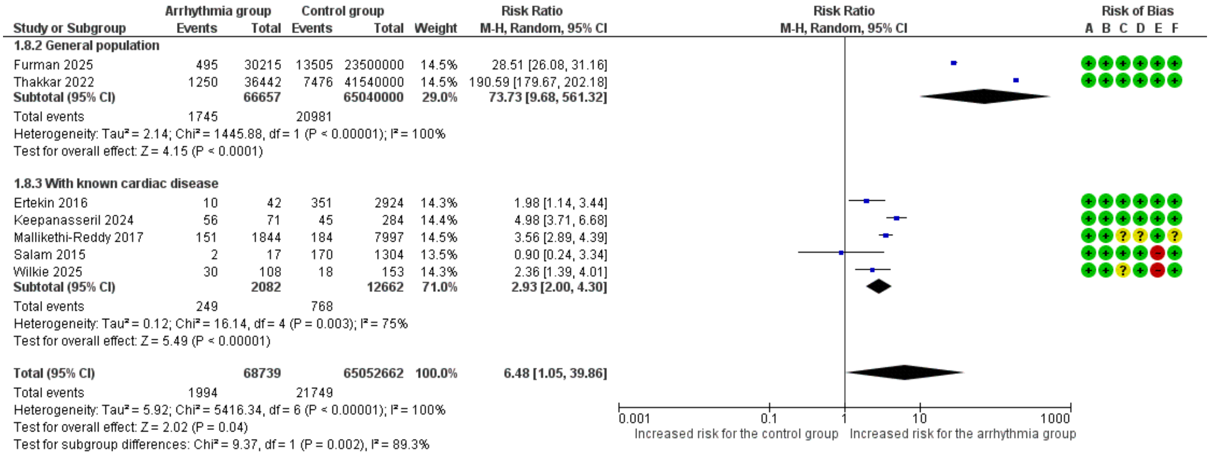

Supplementary Figure S5. Forest Plot of the Subgroup Analysis by Underlying Cardiac Disease for the Outcome of Heart Failure.

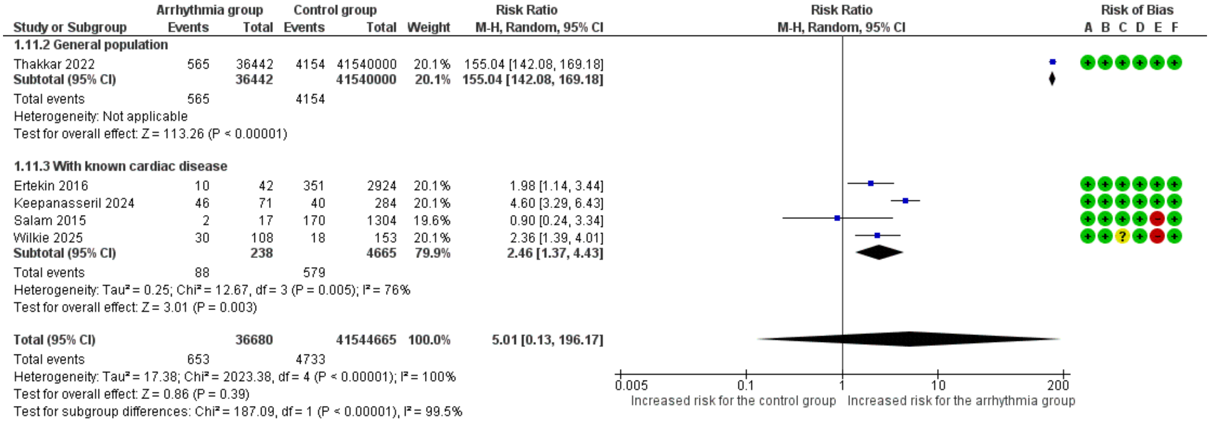

Supplementary Figure 6. Funnel plot and Egger's regression test result for MACE.

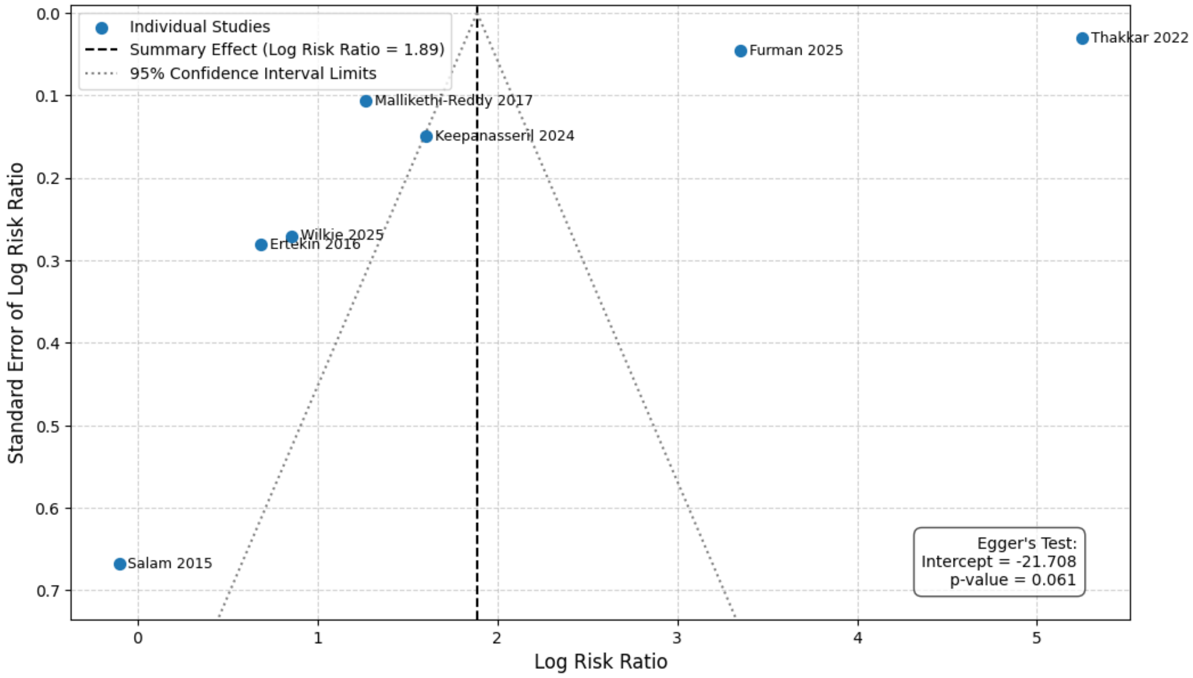

Supplement: Supplementary file 1 [file life-16-00278-s001.zip › Suppl Figures.pdf]
