# Supplementary material for: Impact of Cardiac Arrhythmias on Acute Maternal Cardiovascular Outcomes in Pregnancy: A Systematic Review and Meta-Analysis
Source: Life (Basel). 2026 Feb 5;16(2):278. doi: 10.3390/life16020278 (PMC12941843; doi:10.3390/life16020278)
Supplement: Supplementary file 1 [file life-16-00278-s001.zip › Suppl Tables.pdf]

Supplementary Table S1. The rest of the characteristics for the included studies.

Study	Recurrent or new onset arrhythmia	Adjustment for confounding variables	Antiarrhythmic medication	Anticoagulants	Ablation	Comorbidities
**Bekiaridou et al, 2024 [16]**	Of 76 women with an SVT event, 58 (76.3%) had a prior diagnosis (recurrent) and 18 (23.7%) were new-onset	Multivariable logistic regression adjusted for age and parity.	14% of patients had a history of using beta-blockers, CCBs, or digoxin; 5.7% had a history of Class IC/III agents. For acute SVT during pregnancy, adenosine was used in 11 women; beta-blockers and other AADs were also initiated/reinitiated.	NR	Not performed during pregnancy. Ablation was reported as performed prior to (31 patients) and after (11 patients) pregnancy.	Hypertension: 3.9% (3 out of 76) in the SVT group and 3.0% (9 out of 304) in the control group.<br><br>Diabetes Mellitus: 7.9% (6 out of 76) in the SVT patients and 3.3% (10 out of 304) in controls<br><br>Thyroid Disorder: 10.5% (8 out of 76) in the SVT group and 1.0% (3 out of 304) in controls
**Chou et al, 2023 [17]**	Defined as a condition of frequent PVCs (burden ≥1% on Holter), implying a recurrent/ongoing state rather than a single event.	Propensity score matching (2:1) for age, body mass index, hypertension, diabetes mellitus, thyroid status, parity, meds, delivery type, and echo parameters. Logistic regression post-matching.	No antiarrhythmic drugs (AADs) were used by the PVC group during pregnancy.	NR	Not performed during pregnancy. Three patients underwent ablation after delivery.	DM: 11.5% in PVC group vs. 5.9% in controls<br><br>Hyperthyroidism: 10.8% in the PVC group vs. 2.4% in controls<br><br>Hypertension: 2.9% in the PVC group vs. 3.1% in controls<br><br>Mean BMI: 22.5 ± 4.3 kg/m2 in the arrhythmia group vs. 22.2±3.8 in the control group
**Ertekin et al, 2016 [6]**	Documents the onset of VTA episodes during pregnancy. Does not differentiate between new-onset vs. recurrent	NR	74% of VTA patients used medication during pregnancy; this included beta-blockers (57%),	LMWH (anticoagulant) use was documented in one patient.	NR	Hypertension: In the VTA group, the prevalence was 4.9% whereas in the control group it was 6.5%

	based on pre-pregnancy arrhythmia history.		other AADs (12%), and amiodarone (3 patients).			
**Furman et al, 2025 [18]**	NR	Multivariable logistic regression adjusted for age, race/ethnicity, sex, comorbidity index, hospital region, income, and insurance.	NR	NR	Performed in 190 of 30,215 (0.63%) women during their hospitalization for SVT.	Obesity: 16.1% in the SVT group compared to 10.5% of the controls<br><br>Hypertension: 5.9% of the SVT group versus 1.6% of controls<br><br>DM: 3.4% of the SVT group compared to 1.4% in controls<br><br>Hypothyroidism: 5.9% in the SVT group versus 3.8% in controls<br><br>CHF: 4.5% of the SVT group compared to 0.16% in controls.<br><br>VHD: 4.7% of the SVT group versus 0.24% of controls.<br><br>AF: 3.5% of the SVT group compared to 0.05% in controls<br><br>Previous MI: in 0.36% of the SVT group versus 0.03% of controls
**Keepanasseril et al, 2024 [7]**	47.9% of cases were new-onset AF/AFL during pregnancy. Among 36 women with pre-existing AF/AFL, 13 had a recurrence.	Propensity score matching for maternal age, timing of diagnosis of VHD, severity of mitral stenosis, severity of mitral regurgitation,	Most patients were on beta-blockers (73%) and digoxin (54.5%) at their first visit. All were on beta-blockers after an AF/AFL episode, with some requiring verapamil.	Two-thirds of the AF/AFL group received anticoagulation (heparins, warfarin).	Not mentioned.	Pre-gestational DM: the prevalence was 0% (0 out of 71 patients) in the AF group and 0.6% (8 out of 1322 patients) in controls<br><br>Hypothyroidism: 10.0% in the AF group and 10.1% in controls

		history of heart failure or stroke, cardiac intervention performed before pregnancy.				Hyperthyroidism: 0% in the AF group, compared to 0.7% in controls<br><br>History of HF: 16.9% in the AF group vs. 2.3% in controls
**Mallikethi-Reddy et al, 2017 [19]**	NR	Mixed-effect logistic model adjusted for patient- and hospital-level factors (age, race, comorbidities, income, hospital characteristics).	NR	NR	Catheter ablation was performed in 1.9% of hospitalizations for PPCM with arrhythmias. Electrical cardioversion was performed in 0.3% of this group.	Electrolyte Imbalance affected 29.6% of patients vs. 19.1% of those without arrhythmias<br><br>Obesity: 16.4% in arrhythmia group at compared to 13.8% in the group without arrhythmias<br><br>VHD: 24.2% in the arrhythmia group vs. 16.5% of those without arrhythmias<br><br>Anaemia was lower in the arrhythmia cohort, affecting 67.7% of patients vs. 74.1% of patients without arrhythmias<br><br>Hypertension: 31.3% of patients, compared to 34.7% of patients without arrhythmias
**Salam et al, 2015 [9]**	Of 17 patients with AF/AFL, 7 had a history of arrhythmia before pregnancy (recurrent), and 10 were new-onset.	NR	82% of AF/AFL patients were on medication, including sotalol, digoxin, atenolol, metoprolol, and amiodarone.	Most patients received anticoagulation, including LMWH, UH, OAC, and aspirin.	Not mentioned. The authors note that ablation for AF/AFL during pregnancy had not been reported at the time of the study.	Hypertension was 0% in the Atrial Fibrillation/Flutter (AF/AFL) group, compared to 6.7% (87 out of 1,304 patients) in the control group<br><br>HF: 18% (3 out of 17 patients) in the AF/AFL group vs. 10% (130 out of 1,304 patients) in controls<br><br>AF/AFL before pregnancy: 11.8% (2 out of 17 patients) in the AF/AFL group vs. 1.8% (23 out of 1,304 patients) in the control group

**Siochi et al, 2024 [20]**	Both chronic and new onset AF/AFL was included	Adjusted for patient and hospital-level baseline characteristics.	NR	NR	NR	The study quantified the overall burden of pre-existing conditions using the Charlson Comorbidity Index (CCI), a standardized scoring system, rather than reporting the prevalence of individual comorbidities.
**Thakkar et al, 2022 [8]**	NR	NR	NR	NR	NR	Obesity: 16.95% of the arrhythmia group vs. 8.31% in controls<br><br>Hypertension: 6.91% of the arrhythmia group compared to 1.54% in controls<br>Type 2 DM: 1.39% of the arrhythmia group vs. 0.64% in controls<br>Hypothyroidism: 4.02% in the arrhythmia group vs. 2.06% in controls<br>Hyperlipidaemia: 0.35% of the Arrhythmia group compared to 0.06% in controls<br>HF: 3.02% in the arrhythmia group vs. 0.04% in controls<br>VHD: 4.09% in the arrhythmia group vs. 0.13% in controls<br>Previous MI: 0.22% in the arrhythmia group compared to only 0.01% in controls<br>PVD: 0.23% of the arrhythmia group vs. 0.01% in controls
**Tong et al, 2018 [21]**	Documents specific arrhythmic events during pregnancy. In the PVC group, 2 had sustained VT. In the SVT group, 38% had an SVT episode.	Age-matched groups	In the PVC group, 47% were treated with beta-blockers (metoprolol, bisoprolol, sotalol); two received IV procainamide for VT.	In the SVT group, 2% were on aspirin.	Not performed during pregnancy. One patient had a pre-pregnancy VT ablation; another had a	Hypertension: 2% in the PVC group vs. 4% in the SVT control group, and 0% in the normal control group.<br><br>DM: 4% in the PVC group, and 0% in both the SVT and normal control groups.

					postpartum VT ablation.	Hypothyroidism: 7% in the PVC group, compared to 2% in the SVT control group and 6% in controls
**Wilkie et al, 2025 [22]**	NR	NR	31.5% of the arrhythmia group were on an AAD (e.g., beta-blockers).	NR	Reported in <10 patients, but the timing (before, during, or after pregnancy) is not specified.	NR

Abbreviations: AADs, antiarrhythmic drugs; AF, atrial fibrillation; AFL, atrial flutter; CCBs, calcium channel blockers; CHF: Congestive Heart Failure; DM: diabetes mellitus; IV, intravenous; LMWH, low molecular weight heparin; MI, myocardial infarction; N, number; OAC, oral anticoagulant; PPCM, peripartum cardiomyopathy; PSVT, paroxysmal supraventricular tachycardia; PVC, premature ventricular complex; SVT, supraventricular tachycardia; UH, unfractionated heparin; VA, ventricular arrhythmia; VHD, valvular heart disease; VTA, ventricular tachyarrhythmia; VT, ventricular tachycardia; WPW, Wolff-Parkinson-White syndrome

Reference

1. Bekiaridou, A.; Coleman, K.M.; Sharma, N.; George, D.; Liu, Y.; Gianos, E.; Rosen, S.; Mieres, J.H.; Mountantonakis, S.E. Clinical Significance of Supraventricular Tachycardia During Pregnancy in Healthy Women. *JACC Clin. Electrophysiol.* **2024**, *10*, 1304–1312. https://doi.org/10.1016/j.jacep.2024.03.014.
2. Chou, C.C.; Lee, H.L.; Wo, H.T.; Chang, P.C.; Chiang, C.Y.; Chiu, K.P.; Liu, H.T. Obstetric and fetal/neonatal outcomes in pregnant women with frequent premature ventricular complexes and structurally normal heart. *Int. J. Cardiol.* **2023**, *371*, 160–166. https://doi.org/10.1016/j.ijcard.2022.10.011.
3. Furman, B.W.; Huang, J.; Bhatia, N.K.; Westerman, S.B.; El-Chami, M.F.; Merchant, F.M.; Jain, V. Outcomes of Maternal Supraventricular Tachycardia During Pregnancy. *J. Cardiovasc. Electrophysiol.* **2025**, *36*, 1676–1679. https://doi.org/10.1111/jce.16730.
4. Ertekin, E.; van Hagen, I.M.; Salam, A.M.; Ruys, T.P.; Johnson, M.R.; Popelova, J.; Parsonage, W.A.; Ashour, Z.; Shotan, A.; Oliver, J.M.; et al. Ventricular tachyarrhythmia during pregnancy in women with heart disease: Data from the ROPAC, a registry from the European Society of Cardiology. *Int. J. Cardiol.* **2016**, *220*, 131–136. https://doi.org/10.1016/j.ijcard.2016.06.061.
5. Keepanasseril, A.; Pande, S.N.; Suriya, Y.; Baghel, J.; Mondal, N.; Pillai, A.A.; Satheesh, S.; Siu, S.C. Comparing the outcomes of rheumatic heart disease in pregnancy complicated with and without atrial fibrillation: A propensity score matched analysis. *Am. Heart J.* **2024**, *273*, 140–147. https://doi.org/10.1016/j.ahj.2024.04.002.
6. Thakkar, A.; Kwapong, Y.A.; Patel, H.; Minhas, A.S.; Vaught, A.J.; Gavin, N.; Zakaria, S.; Blumenthal, R.S.; Wu, K.C.; Chrispin, J.; et al. Temporal trends of arrhythmias at delivery hospitalizations in the United States: Analysis from the National Inpatient Sample, 2009–2019. *Front. Cardiovasc. Med.* **2022**, *9*, 1000298. https://doi.org/10.3389/fcvm.2022.1000298.

7. Salam, A.M.; Ertekin, E.; Van Hagen, I.M.; Al Suwaidi, J.; Ruys, T.P.E.; Johnson, M.R.; Gumbiene, L.; Frogoudaki, A.A.; Sorour, K.A.; Iserin, L.; et al. Atrial fibrillation or flutter during pregnancy in patients with structural heart disease data from the ROPAC (Registry on Pregnancy and Cardiac Disease). *JACC Clin. Electrophysiol.* **2015**, *1*, 284–292. https://doi.org/10.1016/j.jacep.2015.04.013.
8. Mallikethi-Reddy, S.; Akintoye, E.; Trehan, N.; Sharma, S.; Briasoulis, A.; Jagadeesh, K.; Rubenfire, M.; Grines, C.L.; Afonso, L. Burden of arrhythmias in peripartum cardiomyopathy: Analysis of 9841 hospitalizations. *Int. J. Cardiol.* **2017**, *235*, 114–117. https://doi.org/10.1016/j.ijcard.2017.02.084.
9. Siochi, C.; Segura Torres, D.; Cervantes, W.; Rabadi, M.; Machado Carvalhais, R.; Sobieraj, P.; Jesmajian, S. Atrial Fibrillation Impacts Inpatient Mortality, Length of Stay, Resource Utilization, Blood Transfusion, and Endotracheal Intubation in Cesarean Sections, Natural Spontaneous Deliveries, and Instrumental Deliveries: A Nationwide Analysis (2016–2020). *Cureus* **2024**, *16*, e74039. https://doi.org/10.7759/cureus.74039.
10. Tong, C.; Kiess, M.; Deyell, M.W.; Qiu, M.; Orgad, M.; Rychel, V.; Claman, A.; Hardwick, E.; McCarthy, B.; Silversides, C.K.; et al. Impact of frequent premature ventricular contractions on pregnancy outcomes. *Heart* **2018**, *104*, 1370–1375. https://doi.org/10.1136/heartjnl-2017-312624.
11. Wilkie, G.L.; Patel, R.; Kovell, L.; Whelan, A. Maternal Cardiac and Perinatal Outcomes Among Pregnant Individuals with Ebstein's Anomaly. *Am. J. Cardiol.* **2025**, *256*, 45–48. https://doi.org/10.1016/j.amjcard.2025.07.025.
